# The Complete Genome Sequence of *Natrinema* sp. J7-2, a Haloarchaeon Capable of Growth on Synthetic Media without Amino Acid Supplements

**DOI:** 10.1371/journal.pone.0041621

**Published:** 2012-07-23

**Authors:** Jie Feng, Bin Liu, Ziqian Zhang, Yan Ren, Yang Li, Fei Gan, Yuping Huang, Xiangdong Chen, Ping Shen, Lei Wang, Bing Tang, Xiao-Feng Tang

**Affiliations:** 1 College of Life Sciences, Wuhan University, Wuhan, Hubei, People's Republic of China; 2 TEDA School of Biological Sciences and Biotechnology, Nankai University, Tianjin, People's Republic of China; 3 The Key Laboratory of Molecular Microbiology and Technology, Ministry of Education, Tianjin, People's Republic of China; University of Florida, United States of America

## Abstract

*Natrinema* sp. J7-2 is an extreme haloarchaeon capable of growing on synthetic media without amino acid supplements. Here we report the complete genome sequence of *Natrinema* sp. J7-2 which is composed of a 3,697,626-bp chromosome and a 95,989-bp plasmid pJ7-I. This is the first complete genome sequence of a member of the genus *Natrinema*. We demonstrate that *Natrinema* sp. J7-2 can use gluconate, glycerol, or acetate as the sole carbon source and that its genome encodes complete metabolic pathways for assimilating these substrates. The biosynthetic pathways for all 20 amino acids have been reconstructed, and we discuss a possible evolutionary relationship between the haloarchaeal arginine synthetic pathway and the bacterial lysine synthetic pathway. The genome harbors the genes for assimilation of ammonium and nitrite, but not nitrate, and has a denitrification pathway to reduce nitrite to N_2_O. Comparative genomic analysis suggests that most sequenced haloarchaea employ the TrkAH system, rather than the Kdp system, to actively uptake potassium. The genomic analysis also reveals that one of the three CRISPR loci in the *Natrinema* sp. J7-2 chromosome is located in an integrative genetic element and is probably propagated via horizontal gene transfer (HGT). Finally, our phylogenetic analysis of haloarchaeal genomes provides clues about evolutionary relationships of haloarchaea.

## Introduction

Haloarchaea thrive in extremely saline environments such as solar salterns, salt lakes, and deposits. These extremophiles have been widely studied because they are readily cultured and have unique features in terms of genetics, phylogeny, physiology, and ecology. Earlier studies on haloarchaea revealed some mechanisms of adaptation to extremely saline environments, i.e. a high intracellular salt concentrations and acidic proteome [1], [2], [3]. Recent advances in haloarchaeal genomics have greatly improved our understanding of the adaptation mechanisms of haloarchaea. The genomic analysis of *Halobacterium salinarum* NRC-1, the first haloarchaeon sequenced, provided new information on the mechanisms by which haloarchaea adapt to saline environments, including high GC content, a large number of ion transporters and signal transduction pathways [4]. The next haloarchaeon sequenced, *Haloarcula marismortui*, was analyzed by comparative genomics methods providing further support for proposed general characteristics of haloarchaea such as an acidic proteome and multiple replicons [5]. The genome of the haloalkaliphilic *Natronomonas pharaonis* provided insights into adaptation to haloalkaline environments [6]. Analyses of the *Nmn. pharaonis* and *Hbt. salinarum* NRC-1 secretome showed the extensive use of the twin-arginine-translocation (Tat) pathway in haloarchaea [6], [7], [8], and the importance of Tat pathway has been experimentally confirmed in *Haloferax volcanii* [9], [10]. To date, at least 35 genera of haloarchaea have been proposed according to the LPSN (List of Prokaryotic names with Standing in Nomenclature, http://www.bacterio.cict.fr/) [11], and 15 complete genomes from 13 genera (*Halobacterium* [4], [12], *Haloarcula* [5], [13], *Halomicrobium* [14], *Haloquadratum* [15], *Halorhabdus* [16], *Halorubrum* [17], *Haloterrigena* [18], *Natronomonas* [6], *Haloferax* [19], *Halogeometricum* [20], *Natrialba*, *Halopiger*, *Halalkalicoccus* [21]) are available in the Genomes On Line Database [22]. However, studies on haloarchaeal genomes are still scarce in comparison with studies of bacterial genomes. Among the sequenced haloarchaea, only four (*Haloarcula hispanica* [23], *Hfx. volcanii* [24], *Nmn. pharaonis* [25] and *Halorhabdus utahensis* [26]) have been experimentally confirmed to be capable of growing in synthetic medium without amino acid supplements, and some metabolic pathways remain to be elucidated [27].

The genus *Natrinema* was created in 1998, as a result of reclassification of three *Halobacterium* species [28]. Six species of the genus *Natrinema*, isolated from salted hide and cod [28], fish sauce [29], and salt lakes [30], [31], [32], are currently recognized. Until this work, there was no complete genome sequence reported for this genus. We previously characterized a plasmid [33], [34], an extracellular protease [35], [36], and a Hsp70 protein [37], [38] of *Natrinema* sp. J7, which was isolated from a salt mine in China. Recently, we found that this haloarchaeon could grow on a synthetic medium without amino acid supplements, making it ideal for analysis of carbohydrate metabolism and amino acid biosynthesis. Here we report the complete genome sequence of *Natrinema* sp. J7-2 (a subculture of strain J7 lacking pHH205) and experiments that investigate aspects of metabolism of this strain, with a focus on carbon and nitrogen metabolism and amino acid synthetic pathways. Interesting findings regarding CRISPR/Cas system and ion transporter are also presented. As the first complete genome of the genus *Natrinema*, these data are of phylogenetic and evolutionary significance.

## Results and Discussion

### 1. General features of the *Natrinema* sp. J7-2 genome

The genome of strain J7-2 is composed of a 3,697,626-bp chromosome and a 95,989-bp plasmid designated pJ7-I. The average GC content of the chromosome is higher than that of pJ7-I (Table 1). The genome has a high percent of protein-coding sequence (∼85.6%) with 4302 predicted protein-coding genes. Approximately 80% of the CDS had a match to proteins in the NCBI nr database. The genome harbors 46 tRNA genes and 10 rRNA genes in three complete rRNA operons, and one of the operons possesses two copies of 5S rRNA genes. In addition, 54 insertion sequences (IS) are present in the chromosome, and approximately half of them (25 IS elements) belong to the IS6 family.

**Table 1:** General information of the *Natrinema* sp. J7-2 genome.

	Chromosome	pJ7-I
Replicon size (bp)	3,697,626	95,989
%GC content	64	59
Percent coding	85.8	77.71
Number of CDSs	4239	63
IS elements a	54	-
Average protein length (amino acid)	249.5	394.7
Number of tRNA b	46	-
Average pI of proteins	5.34	4.44

a The IS elements were gained by Blast analysis of all CDSs against IS DataBase [82].

b The tRNA genes were predicted by tRNAscan-SE with archaeal model [83].

All the genes previously cloned from strain J7, including those encoding proteases SptA and SptB (AY800382), protease SptC (DQ137266), 16S rRNA (DQ874620), GrpE, DnaK and DnaJ (DQ874621), chitinase (EU327592), and bacteriorhodopsin (EU327591), are almost identical to those harbored by strain J7-2 genome (∼99.9% identity). In addition, strain J7-2 does not contain an integrated pHH205 on its genome, and is most likely derived from strain J7 via plasmid loss during long-term storage and successive subculturings.

A draft genome of *Natrinema pellirubrum* DSM 15624 (AGIN01000000), including 64 contigs, had been submitted to GenBank by DOE Joint Genome Institute. Gene prediction performed by us indicated that there are 4555 predicted genes in that draft genome. Genome comparison of strain J7-2 and *Nnm. pellirubrum* DSM 15624 revealed that about 65% of predicted genes (2817) of strain J7-2 have counterparts in *Nnm. pellirubrum* DSM 15624, whereas 1485 predicted genes are only present in J7-2.

### 2. Carbon source metabolism

**Figure 1:**
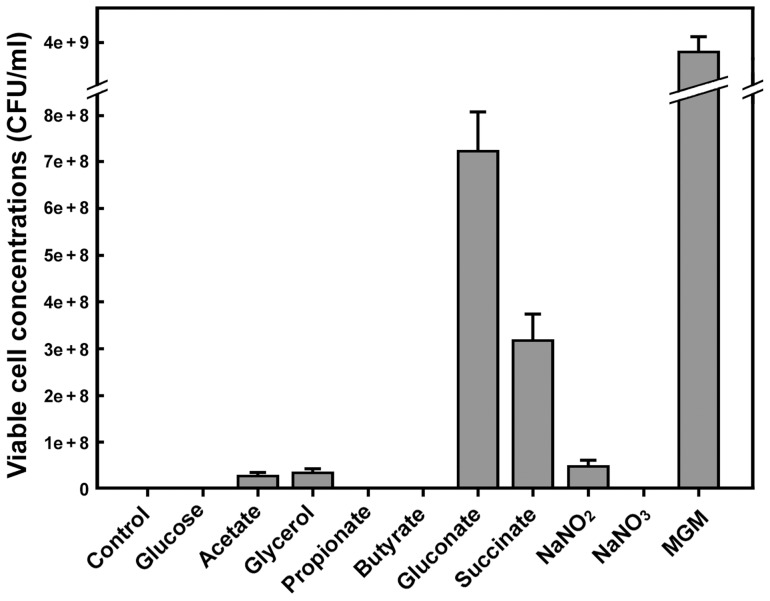
Growth of *Natrinema* sp. J7-2 with different carbon or nitrogen sources. *Natrinema* sp. J7-2 was cultured at 37°C for 7 days in liquid MGM or in liquid synthetic media without (control) or with different carbon or nitrogen sources as indicated. The number of viable cells in each culture was determined on solid MGM as described in the Materials and methods.

**Figure 2:**
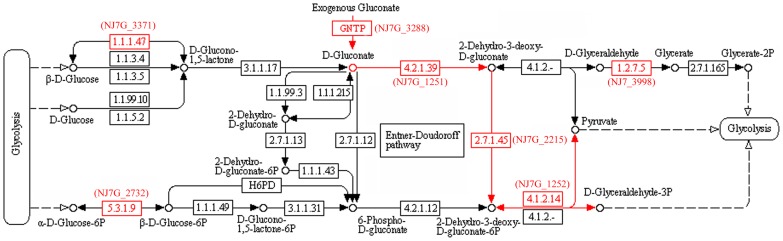
Overview of some carbohydrate metabolic pathways of *Natrinema* sp. J7-2. The metabolic network was reconstructed by KEGG Automatic Annotation Server (KAAS) method. Enzymes identified in *Natrinema* sp. J7-2 are shown in a *red* font, and their gene numbers are given in parentheses. The semi-phosphorylative Entner-Doudoroff (ED) pathway is indicated with *red* arrows.

*Natrinema* sp. J7-2 grows well on gluconate, but not glucose, as the sole carbon source (Figure 1). The key enzyme of the Embden-Meyerhoff pathway, 6-phosphofructokinase, is absent in *Natrinema* sp. J7-2 and other haloarchaea; therefore, they may not have the classical glycolysis pathway [27], [39]. Instead, *Natrinema* sp. J7-2 encodes a semi-phosphorylative Entner-Doudoroff (ED) pathway (Figure 2), which is present in all sequenced haloarchaea except *Nmn. pharaonis* [27], [39]. However, *Natrinema* sp. J7-2 lacks the gene encoding gluconolactonase which is required for conversion of glucono-1, 5-lactone to gluconate (Figure 2), even though its genome encodes a putative glucose dehydrogenase (EC: 1.1.1.47, NJ7G_3371) to convert glucose to glucono-1, 5-lactone. This implies that gluconate, rather than glucose, could enter the semi-phosphorylative ED pathway in this haloarchaeon. A search for possible gluconate and glucose transporters in *Natrinema* sp. J7-2 genome revealed a gluconate/proton symporter (GNTP, NJ7G_3288) for gluconate uptake, but no phosphotransferase system (PTS) for glucose transport (Table S1), which is present in the genome of glucose-utilizing *Hfx. volcanii* [19]. A glucose-specific ABC transporter that mediates only anaerobic glucose transport was previously identified in *Hfx. volcanii* [40]. Although three components of a possible ABC transporter (NJ7G_3553–3555, Table S1) of *Natrinema* sp. J7-2 show 56%, 50%, and 48% identities with components of the glucose-specific ABC transporter (HVO1886–1888) in *Hfx. volcanii*, respectively, they have higher identities (62%, 64% and 66%) with components of a putative sulfate/tungstate ABC transporter (Htur2294–2296) of *Haloterrigena turkmenica*. Taken together, these results indicate that *Natrinema* sp. J7-2 harbors all the genes required for assimilation of exogenous gluconate but lacks the ability to use glucose for growth.

Glycerol and acetate are recognized as two major carbon sources in hypersaline environments and have a role in the nutrition of natural communities of haloarchaea [41]. *Natrinema* sp. J7-2 can grow in a synthetic medium with glycerol or acetate as sole carbon source (Figure 1). The J7-2 genome encodes glycerol kinase (EC: 2.7.1.30, NJ7G_1713) and a multi-subunit glycerol 3-phosphate dehydrogenase (EC: 1.1.5.3, NJ7G_1450, 1715–1717), which is required for the conversion of glycerol 3-phosphate to glycerone phosphate for gluconeogenesis. In addition, the gene encoding triosephosphate isomerase (EC: 5.3.1.1, NJ7G_0774) is present in the J7-2 genome, implying glycerone phosphate could convert into glyceraldehyde-3-phosphate for glycolysis in *Natrinema* sp. J7-2. Among the sequenced haloarchaea, only *Halomicrobium mukohataei* has an identifiable glycerol transporter gene adjacent to a glycerol kinase gene. Other haloarchaea with a glycerol kinase gene have an adjacent gene encoding an uncharacterized membrane protein, which has been predicted to be a new type of glycerol transporter [39], [42] and is also present in *Natrinema* sp. J7-2 (NJ7G_1712, Table S1). For acetate assimilation, *Natrinema* sp. J7-2 lacks the glyoxylate cycle but contains a methylaspartate cycle newly identified in *Har. marismortui* and encoded in an operon [43]. The five key genes of the methylaspartate cycle are also located in an operon (NJ7G_3289–3294) in *Natrinema* sp. J7-2, and are arranged in the same order as in *Har. marismortui*. Meanwhile, the J7-2 genome harbors seven acetyl-CoA synthase genes (EC: 6.2.1.1, NJ7G_1887, 1893, 1904, 2839, 3547, 3549, 3794) involved in conversion of acetate into acetyl-CoA, which could enter the methylaspartate cycle for gluconeogenesis or the TCA cycle for energy production. Like other sequenced haloarchaea [39], the *Natrinema* sp. J7-2 genome possesses a putative acetate transporter (NJ7G_3545) adjacent to a universal stress protein A (UspA, NJ7G_3544). Therefore, *Natrinema* sp. J7-2 has the ability to use glycerol or acetate not only for gluconeogenesis and assimilation of cell carbon but also for energy production.

Although *Natrinema* sp. J7-2 could not grow on synthetic medium using propionate as sole carbon source (Figure 1), it harbors the genes encoding the enzymes for converting propionate to succinyl-CoA via methylmalonyl-CoA (methylmalonate pathway): acetyl-CoA synthetase (EC: 6.2.1.1, NJ7G_1887), propionyl-CoA carboxylase (EC: 6.4.1.3, NJ7G_1889), methylmalonyl-CoA epimerase (EC: 5.1.99.1, NJ7G_1205), and methylmalonyl-CoA mutase (EC: 5.4.99.2 NJ7G_3672). Some haloarchaea, such as *Halorubrum lacusprofundi* [39] and *Nmn. pharaonis* [44] can grow on propionate as a sole carbon source. It may be that *Natrinema* sp. J7-2 lacks the ability to transport proprionate, but little is known about propionate transport in haloarchaea. We searched a local BLAST database that contains all putative propionate transporters in the NCBI nr database, but we did not find a putative propionate transporter in the *Natrinema* sp. J7-2 genome. It is speculated that the inability of this haloarchaeon to grow on propionate as exogenous carbon source is most likely due to the lack of a transporter. Nevertheless, the methylmalonate pathway could be used to degrade endogenous propionate derived from odd-numbered fatty acids or other compounds within the cell.

### 3. Amino acid synthesis

**Figure 3:**
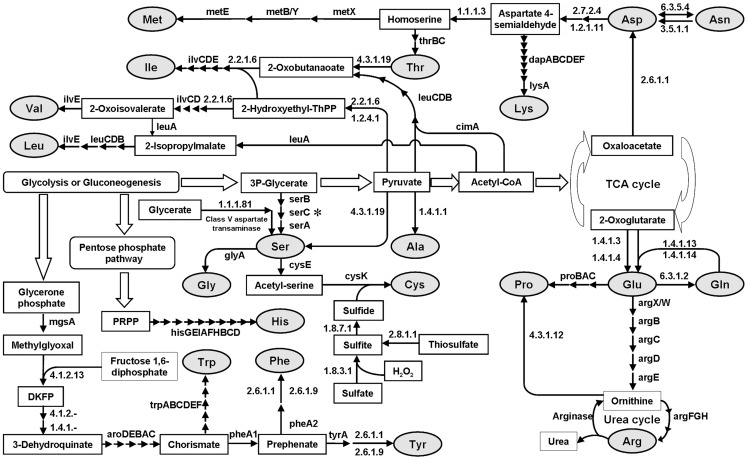
Overview of the amino acid synthesis pathways in *Natrinema* sp. J7-2. The solid arrows depict the enzymatic steps. The open arrows represent the pathways that are encoded by strain J7-2 genome but are not shown in detail here. The missing phosphoserine aminotransferase (*serC*) is indicated by a star. The enzymes (genes) involved in these pathways are listed in Table S2.

*Natrinema* sp. J7-2 can grow on synthetic medium without amino acid supplementation, suggesting *de novo* synthesis of all 20 amino acids. By KEGG orthology assignment and comparative analysis of known haloarchaeal genomes, the amino acid biosynthetic pathways of this haloarchaeon were reconstructed (Figure 3). The *Natrinema* sp. J7-2 genome harbors all the genes required for synthesis of glutamate, glutamine, aspartate, asparagine, alanine, threonine, methionine, valine, and isoleucine (Table S2), and their synthesis pathways have been well depicted elsewhere [23], [27]. Here we focus on the amino acid biosynthetic pathways in which some steps are missing or those that remain to be established in other known haloarchaea [23], [27].

#### Biosynthesis of lysine and arginine

Haloarchaea generally employ the diaminopimelate (DAP) pathway to synthesize lysine from L-aspartate 4-semialdehyde [23], [27]. As shown in Figure 3, the genes (*dapABCDEF* and *lysA*) encoding a complete DAP pathway are present in the J7-2 genome (NJ7G_0260, 4045–4047, 4049–4051, Table S2). All sequenced haloarchaea except *Hbt. salinarum* harbor this pathway; although, *dapF* might be replaced by an unknown non-orthologous gene in *Hmc. mukohataei*, *Hrd. utahensis*, *Har. marismortui*, and *Nmn. pharaonis*.

Another pathway for lysine biosynthesis is the α-aminoadipate (AAA) pathway, which was once thought to be only present in fungi and euglena. Recently, the AAA pathway (*lysW, lysX, lysY, lysZ, lysJ and lysK*) was identified in the bacterium *Thermus thermophilus* [45]. We have not found the AAA pathway in *Natrinema* sp. J7-2 or other sequenced haloarchaea using BLAST; however, all haloarchaeal genomes except that of *Hbt. salinarum* contain two homologs of *lysX* and *lysW* localized in the arginine synthesis gene cluster (e.g., NJ7G_0258–0266) (Figure S1). In this gene cluster, the genes NJ7G_0258–0262, 0265 and 0266 are predicted to be involved in arginine synthesis (*argF/E/D/B/C/H/G*), while NJ7G_0263 and 0264 have been respectively annotated (KEGG orthology) as homologs of *lysX* and *lysW*, of them the products are members of bacterial AAA pathway for lysine synthesis [45]. It is unlikely that the homologs of *lysX* and *lysW* are essential for lysine synthesis in haloarchaea, because they all have a complete DAP pathway. In bacteria, arginine synthesis usually begins with the modification of the α-amino group of glutamate by an acetyl group to avoid intramolecular cyclization of intermediates. The reaction is catalyzed by N-acetylglutamate synthase (ArgA) and/or N-acetylornithine acetyltransferase (ArgJ) [45], but both enzymes are missing in haloarchaea [27]. In the AAA pathway of *T. thermophilus*, LysX activates the γ-carboxyl group of Glu54 of LysW, and then the amino group of α-aminoadipate attaches to this activated group [45]. Because the structure of glutamate is very similar to that of α-aminoadipate (differing only by having an extra methylene), it is reasonable to postulate that the modification of the α-amino group of glutamate in strain J7-2 could be mediated by the products of NJ7G_0263 and NJ7G_0264 (named *argX* and *argW*, respectively), the homologs of bacterial *lysX* and *lysW* (Figure 3 and Figure S1). All the enzymes in the AAA pathway of *T. thermophilus* (LysX, LysZ, LysY, LysJ, and LysK) have basic regions surrounding the active sites to interact with LysW acidic surface [45]. We compared the haloarchaeal enzymes involved in arginine synthesis (ArgB, ArgC, ArgD, and ArgE) with their bacterial homologs and found that similar basic regions are present in the haloarchaeal enzymes but are absent in their bacterial homologs (data not shown). Furthermore, the five-residue C-terminal sequence (EDWGE) of *T. thermophilus* LysW, including the residue Glu54 that is attached to the amino group of aminoadipate [45], is conserved in haloarchaeal ArgW (data not shown). Therefore, it is likely that haloarchaeal enzymes involved in arginine synthesis employ similar reaction mechanisms to their bacterial counterparts in the AAA pathway, and there is an evolutionary relationship between the two pathways.

#### Biosynthesis of praline

Three possible proline biosynthesis pathways have been described for haloarchaea [27]. Proline can be derived from ornithine catalyzed by ornithine cyclodeaminase (EC: 4.3.1.12), homologs of which are encoded in all known haloarchaeal genomes. The second pathway is encoded by the *proABC* gene cluster, wherein proline is generated from glutamate catalyzed successively by glutamate-5-kinase (ProB), glutamate-5-semialdehyde dehydrogenase (ProA), and pyrroline-5-carboxylate reductase (ProC). This pathway is present in the genomes of *Nmn. pharaonis* and *Haloquadratum walsbyi*, but not in *Hbt. salinarum* and *Har. marismortui* [27]. The third pathway, mediated by 1-pyrroline-5-carboxylate dehydrogenase (EC: 1.5.1.12) and proline dehydrogenase (EC: 1.5.99.8, PutA), has also been suggested for haloarchaea based on the evidence that the phylogenetic profile of *putA* is complementary to that of the *proABC* cluster, i.e., *putA* is encoded in *Hbt. salinarum* (OE3955F) and *Har. marismortui* (rrnAC2471), but is missing in *Nmn. pharaonis* and *Hqr. walsbyi* [27]. We found that the *Natrinema* sp. J7-2 genome not only harbors both the ornithine cyclodeaminase gene (NJ7G_0781) and the *proABC* gene cluster (NJ7G_2084–2086), but also encodes proline dehydrogenase (NJ7G_1955, Table S2), indicating that *putA* is unlikely to play a complementary role in *Natrinema* sp. J7-2. It is well known that proline dehydrogenase can participates in proline oxidation, and plays an important role in cellular redox control [46]. It seems likely that *Natrinema* sp. J7-2 employs the first two pathways for proline synthesis, whereas the putative proline dehydrogenase may be involved in proline catabolism. In support of this role for *putA*, a sodium:proline symporter (NJ7G_1953, Table S1) for proline uptake is encoded downstream of *putA* in *Natrinema* sp. J7-2.

#### Biosynthesis of leucine

Like *Natrinema* sp. J7-2, *Nmn. pharaonis* is able to grow on a synthetic medium without amino acid supplements; however, the *leuA* gene encoding isopropylmalate synthase (NP2206A) involved in the biosynthesis of leucine is interrupted in the 5′-region, and the reason why this does not result in leucine auxotrophy is uncertain [25]. The strain J7-2 genome possesses a complete pathway for leucine biosynthesis (Figure 3, Table S2), including an intact *leuA* gene (NJ7G_1743).

#### Biosynthesis of histidine

*Natrinema* sp. J7-2 harbors a set of genes (*hisG/E/I/A/F/H/B/C/D*) needed for histidine biosynthesis (Figure 3, Table S2). In contrast to *hisB* of *Escherichia coli* [47], which encodes a bifunctional protein composed of two domains (imidazoleglycerol-phosphate dehydratase and histidinol-phosphatase), haloarchaeal *hisB* only encodes the imidazoleglycerol-phosphate dehydratase [27]. In *Natrinema* sp. J7-2, a putative histidinol-phosphatase gene (NJ7G_3071, Table S2) may encode the other function, and homologs are also present in other haloarchaeal genomes (e.g., HQ1692A of *Hqr. walsbyi*).

#### Biosynthesis of serine, glycine and cysteine

In haloarchaea, serine biosynthesis is predicted to be accomplished via a phosphorylated synthesis pathway encoded by three genes, *serA*, *serB,* and *serC* [27], wherein glycerate 3-phosphate is oxidized to 3-phospho-hydroxy-pyruvate, which is subsequently converted to phosphoserine and then to serine. Like all other sequenced haloarchaea, *Natrinema* sp. J7-2 possesses *serA* (NJ7G_0555, 0762, 2895) and *serB* (NJ7G_0559, 4104), but is missing *serC* which encodes the phosphoserine aminotransferase (Figure 3). We constructed a BLAST database containing all known phosphoserine aminotransferases in GenBank, and compared the *Natrinema* sp. J7-2 genome to this database using BLAST at the protein level, but did not find any homologous proteins. Perhaps the function of *serC* has been replaced by an unknown non-orthologous gene in haloarchaea. A possible alternative pathway in *Natrinema* sp. J7-2 is direct derivation of serine from pyruvate catalyzed by serine/threonine dehydratase (EC: 4.3.1.19, NJ7G_2201). Besides, a non-phosphorylated serine synthesis pathway has been predicted in some haloarchaea (e.g., *Nmn. pharaonis*) [27]. Two enzymes involved in the non-phosphorylated pathway, hydroxypyruvate reductase (EC: 1.1.1.81, NJ7G_4038) and class V aspartate transaminases (NJ7G_0571, 2948), were found in the *Natrinema* sp. J7-2 genome (Figure 3). Because *Natrinema* sp. J7-2 does not have threonine aldolase to produce glycine from threonine, glycine seems to be derived only from serine catalyzed by glycine hydroxymethyltransferase encoded by *glyA* (NJ7G_3408, Figure 3).

*Natrinema* sp. J7-2 harbors the genes required for the synthesis of cysteine from serine via acetylserine by transfer of hydrogen sulfide. However, little is known about the biosynthesis of hydrogen sulfide in haloarchaea. The hydrogen sulfide formation pathway from sulfate via adenylylsulfate, 3P-adenylylsulfate and sulfite is absent in *Natrinema* sp. J7-2 and other sequenced haloarchaea [27]. Thiosulfate might be converted to sulfite by thiosulfate sulfurtransferase (NP3186A) and subsequently to sulfide by sulfite reductase (NP4004A) in *Nmn. pharaonis* [27]. The genes encoding these two enzymes (EC: 2.8.1.1, NJ7G_0294; EC: 1.8.7.1, NJ7G_0304) are present in the J7-2 genome (Figure 3). Besides, the J7-2 genome encodes a putative sulfite oxidoreductase (EC: 1.8.3.1, NJ7G_3031), by which sulfate could be directly reduced to sulfite, and the latter would then be reduced to hydrogen sulfide by sulfite reductase (EC: 1.8.7.1, NJ7G_0304, Figure 3). This notion is supported by the finding that *Natrinema* sp. J7-2 can grow on synthetic medium with sulfate as sole sulfur source (see Material and Methods). Sulfite oxidoreductase homologs are also present in other haloarchaea and active site residues are conserved in these putative enzymes (data not shown).

#### Biosynthesis of aromatic amino acids

Phenylalanine, tyrosine and tryptophan are predicted to be synthesized in *Natrinema* sp. J7-2 with 3-dehydroquinate as a common precursor, but the genes encoding the classical shikimate pathway enzymes to synthesize 3-dehydroquinate from erythrose 4-phosphate and phosphoenolpyruvate are missing. In haloarchaea, 3-dehydroquinate might be synthesized from 6-deoxy-5-ketofructose 1-phosphate (DKFP) [27]. In agreement with this, the DKPF pathway enzymes for 3-dehydroquinate synthesis, 2-amino-3, 7-dideoxy-D-threo-hept-6-ulosonate synthase (EC: 4.1.2.-, NJ7G_4134), and 3-dehydroquinate synthase II (EC: 1.4.1.-, NJ7G_4142) are encoded in the *Natrinema* sp. J7-2 genome (Figure 3). As in methanogenic archaea [48], the precursor DKFP might be synthesized from methylglyoxal instead of nucleoside diphosphate sugars in this haloarchaeon (Figure 3). In contrast to *Nmn. pharaonis* which lacks methylglyoxal synthase to synthesize methylglyoxal from glycerone phosphate [27], the *Natrinema* sp. J7-2 genome encodes a methylglyoxal synthase (EC: 4.2.3.3, NJ7G_2096, Figure 3) as do most other sequenced haloarchaea (e.g., rrnAC2162, HQ1527A, etc.). Methylglyoxal and fructose 1, 6-diphosphate (or fructose 1-phosphate) are then used as substrates for multifunctional fructose 1,6-bisphosphate aldolase (EC: 4.1.2.13, NJ7G_3648, Figure 3) to synthesize DKPF [27]. Therefore, *Natrinema* sp. J7-2 harbors complete biosynthetic pathways for aromatic amino acids from glycerone phosphate.

### 4. Nitrogen metabolism

Inorganic ammonium, nitrate and nitrite can be assimilated by some haloarchaea as nitrogen source [49], [50]. Ammonium as the sole nitrogen source can support the growth of *Natrinema* sp. J7-2 (Figure 1). Ammonium could be taken up via a putative transporter AmtB (NJ7G_0366, Table S1), and would be directly incorporated into the carbon skeletons by glutamine synthetase (EC: 6.3.1.2, NJ7G_1637, 2663) and glutamate synthase (EC: 1.4.1.13/14, NJ7G_2607), and glutamate dehydrogenase (EC: 1.4.1.3, NJ7G_1599, 1939, 2174; EC: 1.4.1.4, NJ7G_3635, Table S2). Many haloarchaea can also generate ammonium inside the cell via assimilatory nitrate reductase (Nas) and nitrite reductase (Nir) which reduce nitrate to ammonium via nitrite [49]. However, *Natrinema* sp. J7-2 lacks a *nas* gene, although it harbors two genes encoding ferredoxin-nitrite reductase (EC: 1.7.7.1, NJ7G_0304, 0621). This haloarchaeon is also predicted to encode a formate-nitrite transporter (FNT, NJ7G_3263, Table S1) [39], but lacks nitrate transporters such as NarK, which are encoded by nitrate-utilizing haloarchaea [6], [51]. This may account for the ability of *Natrinema* sp. J7-2 to grow on sodium nitrite rather than sodium nitrate as the sole nitrogen source (Figure 1).

Denitrification is a key process of the N-cycle in nature, whereby nitrate is successively reduced to nitrite, NO, N_2_O and N_2_. Some haloarchaea appear to be capable of denitrification. When cultured anaerobically on nitrate, *Hfx. denitrificans*, *Hfx. mediterranei* and *Har. marismortui* could produce N_2_, implying they have a complete denitrification pathway [5], [52]. In the case of *Natrinema* sp. J7-2, genes encoding two copper-containing respiratory nitrite reductases (Cu-Nir, EC: 1.7.2.1, NJ7G_3094, 2432) and a nitric oxide reductase (NorB, EC: 1.7.99.7, NJ7G_0368) are present, but genes encoding respiratory nitrate reductase (Nar) and nitrous oxide reductase (Nos) are not, indicating that it has an incomplete denitrification pathway to reduce nitrite to N_2_O via NO.

### 5. Transporters and ion channels

More than 60 types of transporters responsible for nutrient uptake, osmotic regulation, cation/anion transport and toxin export were identified in *Natrinema* sp. J7-2 genome (Table S1). Multiple transport systems involved in osmotic regulation were detected, including active potassium transport system (TrkAH), multicomponent K^+^/Na^+^:H^+^ antiporter (Pha/Mnh), and Na^+^:H^+^ antiporter (NhaC) (Table S1). Haloarchaea are known to maintain a high intracellular K^+^ concentration (∼4 M) to cope with hypersaline environments. The genomes of *Hbt. salinarum* NRC-1 and *Hbt. salinarum* R1 possess an ATP-driven K^+^ transport system (KdpABC) in addition to the TrkAH transporter driven by the membrane potential [4], [12]. However, *Natrinema* sp. J7-2 and other sequenced haloarchaea harbor the TrkAH transporter and lack the Kdp system. Recently, the Kdp system has been experimentally verified to be inducible in *Hbt. salinarum* R1, enabling it to grow under extreme K^+^-limiting conditions (>20 μM) [53]. Kdp system deletion strains exhibited a growth curve identical to the wild-type under non-limiting K^+^ concentrations (e.g., 50 mM) [53]. *Natrinema* sp. J7-2 lacking the Kdp system could grow well both in MGM medium with ∼60 mM K^+^ and in a complex medium without K^+^ supplement [35], implying that another active K^+^ transport system (e.g., TrkAH) is functional in this haloarchaeon. Although the role of haloarchaeal TrkAH in K^+^ uptake remains to be experimentally confirmed, the conservation of TrkAH in all sequenced haloarchaea leads us to postulate that haloarchaea mainly employ the TrkAH transporter to actively transport K^+^ into cells.

### 6. Cas/CRISPR system

**Figure 4:**
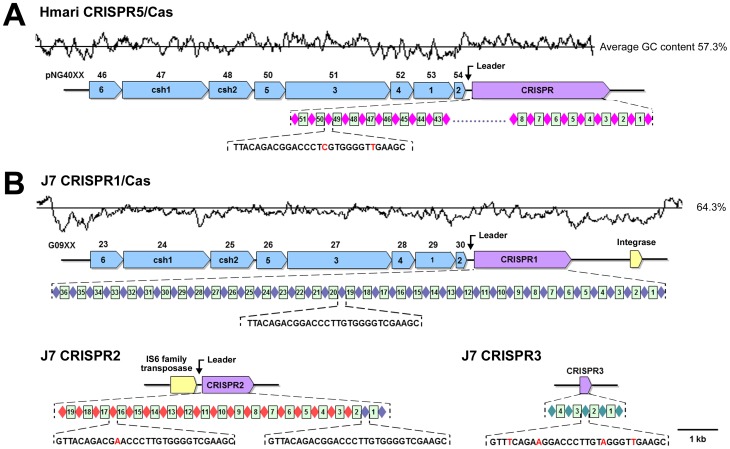
Overview of the CRISPR/Cas systems in pNG400 and *Natrinema* sp. J7-2. (A) The Hmari CRISPR5/Cas system in pNG400. (B) The J7 CRISPR1/Cas system in *Natrinema* sp. J7-2. The Cas genes (*blue*), CRISPRs (*purple*), integrase gene and IS6 family transposase gene (*yellow*) are drawn to scale as arrows. The accession numbers of the Cas genes are shown above the arrows. Diamonds and rectangles represent the direct repeats and spacers, respectively. Direct repeats with identical sequences are shown in the same color. The sequences of the direct repeats are given, and the nucleotides that differ from those of the direct repeat of J7 CRISPR1 are shown in red. The GC content is plotted above the diagrams of the CRISPR/Cas regions, with the average GC content for the region indicated as a straight black line (average % GC is given at the right).

Clustered regularly interspaced short palindromic repeats (CRISPRs) and CRISPR-associated (Cas) genes are widely distributed in bacteria and archaea, and have been described to act as a form of acquired immunity against mobile-genetic invasion by viruses and plasmids [54], [55], [56], [57]. All sequenced haloarchaea except *Hqr. walsbyi* and *Hbt. salinarum* NRC-1 possess CRISPRs [19], [58]. CRISPR loci typically consist of a leader sequence, several noncontiguous direct repeats separated by stretches of variable sequences called spacers and are often adjacent to *cas* genes [57]. Three CRISPR loci were identified in the *Natrinema* sp. J7-2 genome, and named J7 CRISPR1, J7 CRISPR2, and J7 CRISPR3, but they differ from each other in their compositions (Figure 4).

The J7 CRISPR1 locus is composed of a leader sequence, 37 identical repeats and 36 spacers, and is preceded by and a full set of *cas* genes (Figure 4B). The J7 CRISPR1/Cas locus has a high level of sequence identity (85%) to a CRISPR/Cas locus (Hmari CRISPR5/Cas) in the plasmid pNG400 from *Har. marismortui* [5], [58], and the *cas* genes are arranged in the same order (Figure 4). Although their spacer sequences are different, the noncontiguous direct repeats of the two CRISPRs differ only in two sites (Figure 4). The high genetic similarity between the two CRISPR/Cas systems strongly suggests that they are derived from a common ancestor. Interestingly, the J7 CRISPR1/Cas locus appears to be located in an integrative genetic element, because its average GC content (58%) is lower than that of the whole genome (64.3%), and an integrase gene is present at the border of the element (Figure 4B). Similarly, the GC content (57.3%) of pNG400 is also lower than that of the chromosome (62.4%) of *Har. marismortui* [5]. The finding that the two homologous CRISPR loci are both located in mobile genetic elements implies that they might be propagated via horizontal gene transfer [54].

The J7 CRISPR2 locus differs from the J7 CRISPR2 locus in that there is no *cas* gene adjacent to the leader sequence. Instead, an IS6 family transposase gene is present in the vicinity of the leader sequence of J7 CRISPR2, which shares 50% identity to that of J7 CRISPR1 (Figure 4B). Two repeats of J7 CRISPR2 are identical to that of J7 CRISPR1, but the subsequent repeats have a point mutation (Figure 4B). In addition, the spacers of J7 CRISPR1 are not conserved in J7 CRISPR2. It seems that J7 CRISPR2 was derived from J7 CRISPR1, most likely through a transposition process. CRISPR structures devoid of neighboring *cas* genes have been found in other haloarchaea (e.g., *Har. marismortui* and *Nmn. pharaonis*) [56], [58].

In contrast to the J7 CRISPR1 and CRISPR2 loci, the J7 CRISPR3 locus lacks a leader sequence. In addition, *cas* genes were not found in the vicinity of the J7 CRISPR3 locus (Figure 4B). The leader sequence is required for CRISPR-adaptation and expression [57], suggesting that the J7 CRISPR3 is inactive. In addition, the repeat of J7 CRISPR3 differs from that of J7 CRISPR1 at four bp.

Previous studies suggest that CRISPR spacers derive from preexisting sequences, either chromosomal or within transmissible genetic elements such as phages and plasmids [59]. Among the 59 spacers of the three J7 CRISPRs, six show significant similarities to known sequences (Table S3). Interestingly, the spacer 2 of J7 CRISPR2 is closely related to a fragment of pHH205 (28 identities in 30 nt). This might be seen as a record of previous existence or invading of pHH205 in strain J7-2; however, plasmids and phages can circumvent CRISPR-based immunity by mutating the proto-spacer they carry [57]. Therefore, the difference in two sites between the spacer 2 of J7 CRISPR2 and the pHH205 fragment will allow the plasmid to overcome the immunity, even if J7 CRISPR2 is active. Our ongoing experiment, in which we are working on transformation of strain J7-2 with pHH205, is expected to address this issue.

### 7. Phylogenetic analyses

**Figure 5:**
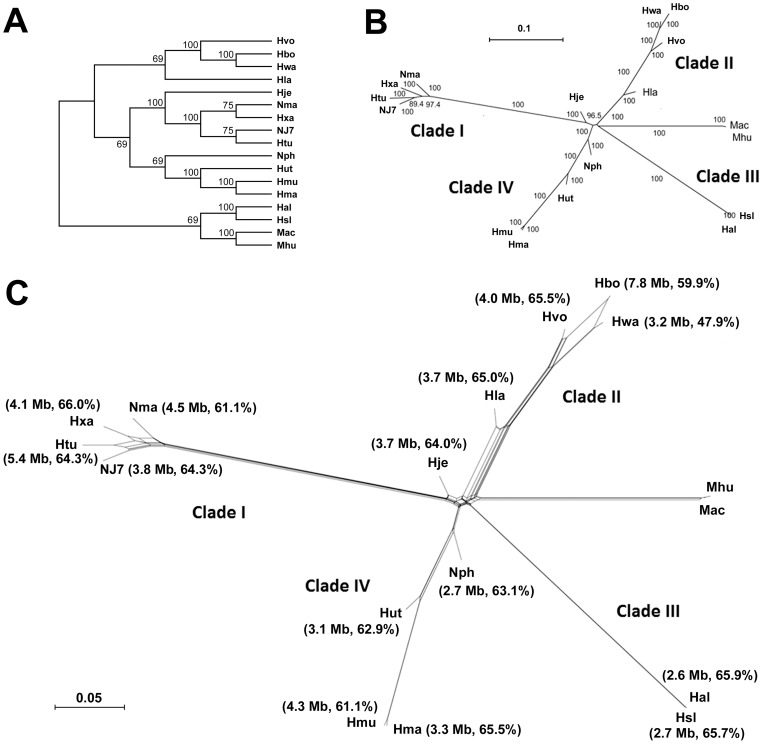
Phylogenomic analyses of haloarchaea. (A) The MSSA supertree of haloarchaea and two outgroup genomes (Mhu and Mac). (B) The Neighbor-Joining phylogeny of haloarchaeal Baum-Ragan pseudo-sequences. The numbers mark the bootstrap values (>70%) of each branch out of 1000 bootstrap resamplings. (C) The phylogenetic Neighbor-Net network of Baum-Ragan pseudo-sequences. The genome size and GC content of each strain are shown in parentheses. Abbreviations: NJ7, *Natrinema* sp. J7-2; Hma, *Har. marismortui* ATCC 43049; Hal, *Hbt. salinarum* R1; Hsl, *Hbt. salinarum* NRC-1; Hmu, *Hmc. mukohataei* DSM 12286; Hwa, *Hqr. walsbyi* DSM 16790; Hut, *Hrd. utahensis* DSM 12940; Hla, *Hrr. lacusprofundi* ATCC 49239; Htu, *Htg. turkmenica* DSM 5511; Nph, *Nmn. pharaonis* DSM 2160; Hvo, *Hfx. volcanii* DS2; Hbo, *Hgm. borinquense* DSM 11551; Nma, *Nab. magadii* ATCC 43099; Hje, *Halalkalicoccus jeotgali* B3; Hxa, *Hpg. xanaduensis* SH-6; Mac, *Methanosarcina acetivorans* C2A; Mhu, *Methanospirillum hungatei* JF-1.

The genomes of 15 sequenced haloarchaea and two outgroup genomes were subjected to phylogenomic analyses using three methods. We selected 851 core gene sets distributed in the 17 genomes to construct maximum likelihood (ML) trees. Then, the ML phylogenies were combined to reconstruct a supertree (Figure 5A) by most similar supertree analysis (MSSA) method [60]. Meanwhile, all the ML phylogenies were combined into pseudo-sequences by the matrix representation method [61], [62], and a phylogenetic tree (Figure 5B) was also reconstructed from the pseudo-sequences by Neighbor-Joining (NJ) method [63]. The two supertrees created using the two methods showed similar topology, and the distribution of the 15 sequenced haloarchaea on the supertrees is coincident with that on the phylogenetic tree based on 16S rRNA gene sequences (Figure S2). According to the phylogenomic analyses, the 15 sequenced haloarchaea appear to form at least four clades, wherein *Natrinema* sp. J7-2 is closely related to *Htg. turkmenica*, *Halopiger xanaduensis* and *Natrialba magadii*. However, the simple branching trees can not show the underlying evolutionary history that is not tree-like, such as changes driven by recombination, gene loss, and HGT which play important roles in microbial evolution. Therefore, by using the Neighbor-Net method [64], [65], the pseudo-sequences were employed to reconstruct a phylogenetic network (Figure 5C) to represent conﬂicting signals or alternative phylogenetic histories in the pseudo-sequences (Baum-Ragan matrix). Generally, the evolutionary relationship between the genomes visualized by the phylogenetic network is similar to that apparent in the phylogenetic trees (Figure 5). However, the phylogenetic network shows possible non-vertical evolution evolutionary history in haloarchaea. For example, obvious non-vertical evolution events have occurred relatively recently among *Haloquadratum*, *Halogeometricum*, and *Haloferax* in clade II. Meanwhile, the vast differences in genome sizes and GC contents between different members of clade II could also potentially reflect frequent gene exchange. In the case of clade I, the presence of the boxes also implies non-vertical evolutionary events have occurred among *Natrinema*, *Haloterrigena*, *Halopiger*, and *Natrialba* (Figure 5C).

### 8. Conclusion

The ability of *Natrinema* sp. J7-2 to grow in synthetic media without amino acid and vitamin supplements makes it ideal for investigation of metabolic pathways in haloarchaea. The genomic analysis of *Natrinema* sp. J7-2 and growth experiments described here enabled us to devise a scheme of the biosynthetic pathways of all 20 amino acids in a haloarchaeon and also provided new clues for carbon and nitrogen metabolism of haloarchaea. Further biochemical and genetic studies are required to test these predictions. In addition, as the first completely sequenced member of *Natrinema* genus, the genome of *Natrinema* sp. J7-2 is of phylogenetic and evolutionary significance, and will help us better understand archaeal biology and the mechanisms by which microorganisms adapt to extreme environments.

## Materials and Methods

### Strains and culture conditions

*Natrinema* sp. J7, previously named as *Halobacterium salinarum* J7 (CCTCC AB91141), was isolated from a salt mine in Hubei province, China in the early 1990s [33], [34]. This strain was stored at room temperature both in lyophilized state and in a form of culture grown on 18% MGM agar plate [35], [36] sealed with Parafilm, and was renewed every 1.5 to 2 years. The strain J7-2, which lacks plasmid pHH205, was occasionally identified in an effort to isolate the plasmid from renewed subcultures of the stored plate cultures of *Natrinema* sp. J7.

*Natrinema* sp. J7-2 cells were grown in liquid 18% MGM [35], [36], and the genomic DNA was prepared according to the method of Kamekura et al. [66]. A synthetic medium, Hv-Min [67] was modified for analysis of nutrition utilization of *Natrinema* sp. J7-2. The modified Hv-Min synthetic medium (per liter) consists of 3.2 M NaCl, 0.17 M MgSO_4_·7H_2_O, 0.33 M KCl, 5 mM CaCl_2_·2H_2_O, 1 ml of trace elements stock solution [68], 60 mM sodium succinate, 0.2 mM NH_4_Cl, and 0.6 mM potassium phosphate buffer (pH 7.0). In some cases, sodium succinate or NH_4_Cl was replaced by equal amount (in mole) of other carbon or nitrogen sources. *Natrinema* sp. J7-2 cells were cultivated aerobically with rotary shaking at 180 rpm at 37°C. The number of viable cells in liquid cultures was determined on 18% MGM agar plates incubated at 37°C for 7 days.

### Genome sequencing and assembly

The genome of *Natrinema* sp. J7-2 was determined by Roche 454 pyrosequencing and Sanger dideoxy sequencing. For Roche 454 pyrosequencing, 362,202 reads with an average length of 213 bp were generated using the Roche GS FLX system, representing a theoretical 20.3-fold genome coverage. For Sanger dideoxy sequencing, a plasmid library of 6–10 kb inserts generated by mechanical shearing of genomic DNA was constructed in pUC118. Double-ended plasmid sequencing reactions were carried out with BigDye terminator chemistry on ABI 3730 capillary sequencers, and 12,673 reads with an average length of 826 bp were generated, providing a theoretical 2.8-fold genome coverage. All sequence data were assembled using the 454/Roche Newbler assembly program, the gaps between contigs were closed by targeted PCR, and PCR products were sequenced on ABI 3730 capillary sequencers.

### Gene prediction and annotation

Open reading frames (ORFs) were identified by using CRITICA [69] and Glimmer [70], followed by BLASTX searches of the remaining intergenic regions. Artemis [71] was used to collate data and facilitate annotation. Function predictions were based on BLASTp similarity searches in the UniProtKB, GenBank, Swiss-Prot protein databases, and the clusters of orthologous groups (COG) database (www.ncbi.nlm.nih.gov/COG). All annotations were inspected manually through searches against PFAM [72], Simple Modular Architecture Research Tool (SMART) [73], and PROSITE [74].

### Metabolic pathway construction

All CDS in *Natrinema* sp. J7-2 genome were searched against the KEGG database by the BBH (bi-directional best hit) method of KAAS (KEGG Automatic Annotation Server) [75] to make KO (KEGG orthology) assignments, which were then used to map the CDS to metabolism pathways with KEGG tools KegHier [75]. Because the KAAS analysis only uses a subset of genomes to make KEGG assignments, some functions for divergent organisms like haloarchaea may be more difficult to identify. For pathways in which some functions were missing or for pathways unique to haloarchaea, related protein sequences were downloaded from the nr database (containing all non-redundant sequences from GenBank CDS translations, PDB, Swiss-Prot, PIR, and PRF) and formatted into a local BLAST database. We then searched the local database for proteins similar to the CDSs in *Natrinema* sp. J7-2 genome using BLAST program [76] to identify additional functions.

### Phylogenetic analyses

The genomes of selected microorganisms were obtained from NCBI genome database (http://www.ncbi.nlm.nih.gov/sites/genome). We searched the groups of orthologous proteins in the selected genomes using the OrthoMCL program [77], and appropriate orthologous gene groups were selected for further analysis. The MAFFT program version 6.833 [78] was employed to align each group using the default settings. Thereafter, the maximum likelihood (ML) phylogeny of each of the gene groups was constructed and estimated with 100 times bootstrap resampling using PhyML program version 3.0 [79]. Custom-made scripts were used to find well-supported (>70% bootstrap support) bipartitions in each gene trees. The two clades that make up the bipartitions are assigned an A or T, and the members of each clade also assume this designation. For every strain subjected to analysis, its A/T designation is extracted for each gene tree and used to build a pseudo-sequence (Baum-Ragan matrix) [61], [62]. The Neighbor-Net method [64] treats each of the substitutions equally and thus the A/T designation will not import systematic bias. All the pseudo-sequences of the selected strains were used to re-construct the phylogenic network using Neighbor-Net [64] and Neighbor-Joining [63] methods implanted in SplitsTree 4.0 [80]. Besides, the best supertree topology was reconstructed with heuristic search of tree space using the most similar supertree method (MSSA) [60] as implemented in Clann version 3.1.3 [81].

### Nucleotide sequence accession number

The genome sequence of *Natrinema* sp. J7-2 has been deposited in GenBank under accession numbers: CP003412 (chromosome) and CP003413 (plasmid pJ7-I).

## Supporting Information

Figure S1 **The arginine synthesis gene clusters of some haloarchaea.** The genes are drawn to scale as arrows.
(TIF)

Figure S2 **Maximum-likelihood phylogenetic tree of 16S rRNA genes of haloarchaea.** The numbers mark the above 50 bootstrap values for each node out of 100 bootstrap resamplings. The sequenced haloarchaea are named as indicated in the legend of Figure 5, and their 16S rRNA gene sequences were obtained from the genomes. The 16S rRNA gene sequences of other haloarchaea were collected from survey studies. Among the three 16S rRNA genes of Natrinema sp. J7-2, two copies show 100% identity to the partial sequence of the 16S rRNA gene of Nnm. gari JCM14663 (AB545859), while the third one differs from the latter at two nucleotides. This indicates Natrinema sp. J7-2 is closely related to Nnm. gari.
(TIF)

Table S1 **Predicted transporters in** ***Natrinema*** **sp. J7-2 genome.**
(DOC)

Table S2 **Predicted enzymes involved in amino acid synthesis in** ***Natrinema*** **sp. J7-2.**
(DOC)

Table S3 **Alignments of some CRISPR spacers of Natrinema sp J7-2 with the most similar known sequences.**
(DOC)
