# Supplementary figures and images for: The Complete Genome Sequence of Natrinema sp. J7-2, a Haloarchaeon Capable of Growth on Synthetic Media without Amino Acid Supplements
Source: PLoS One. 2012 Jul 23;7(7):e41621. doi: 10.1371/journal.pone.0041621 (PMC3402447; doi:10.1371/journal.pone.0041621)

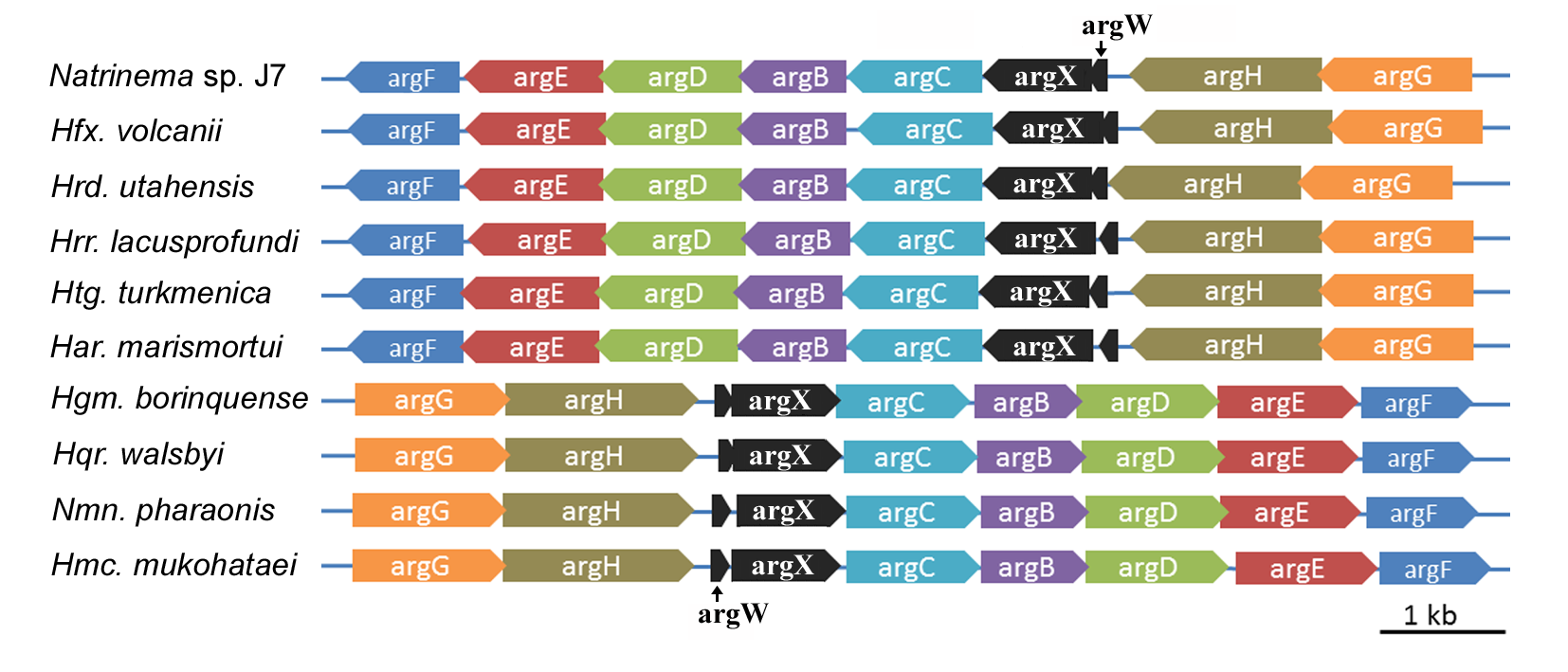

Supplement: Figure S1 — The arginine synthesis gene clusters of some haloarchaea. The genes are drawn to scale as arrows. (TIF) [file pone.0041621.s001.tif]

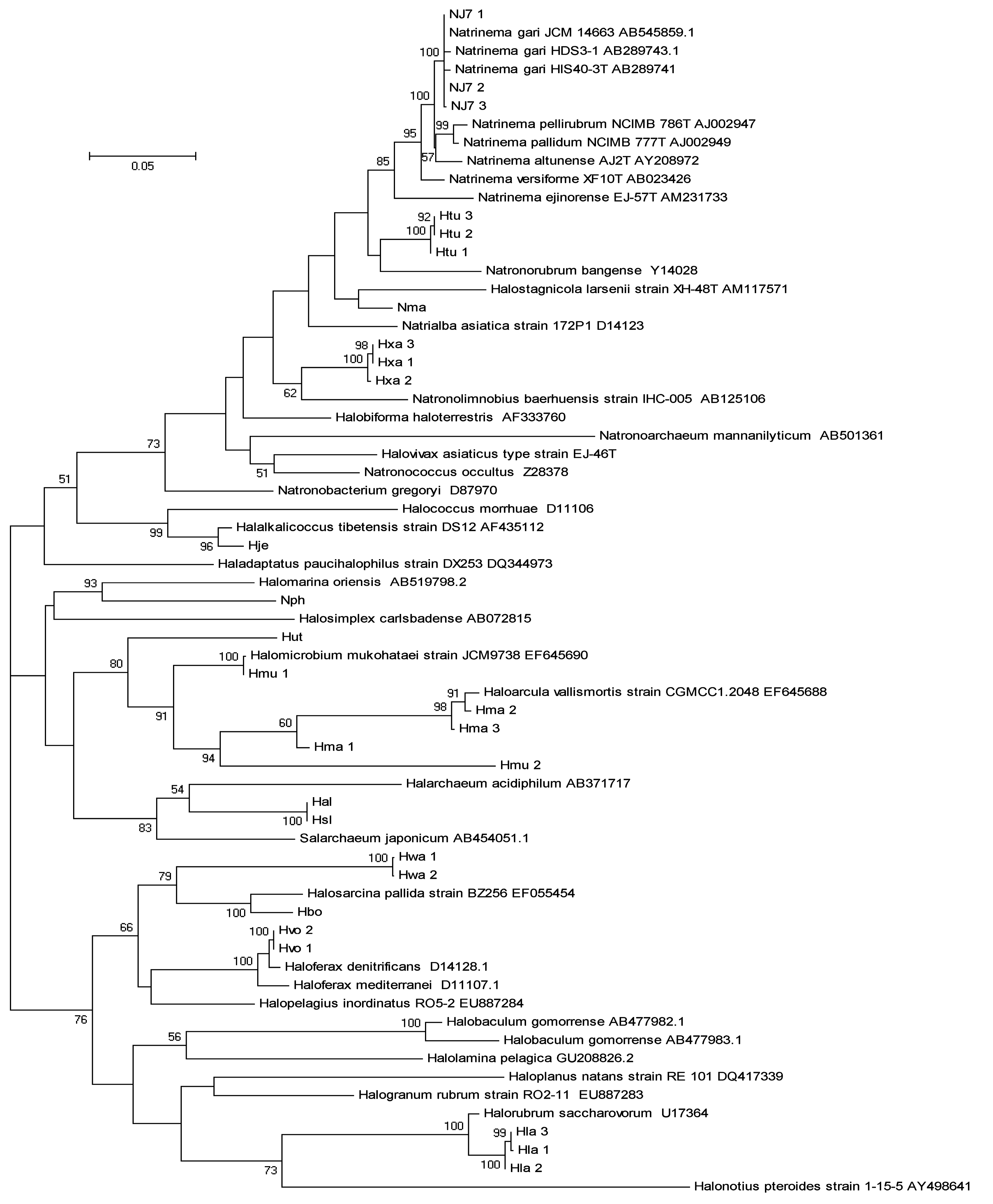

Supplement: Figure S2 — Maximum-likelihood phylogenetic tree of 16S rRNA genes of haloarchaea. The numbers mark the above 50 bootstrap values for each node out of 100 bootstrap resamplings. The sequenced haloarchaea are named as indicated in the legend of Figure 5, and their 16S rRNA gene sequences were obtained from the genomes. The 16S rRNA gene sequences of other haloarchaea were collected from survey studies. Among the three 16S rRNA genes of Natrinema sp. J7-2, two copies show 100% identity to the partial sequence of the 16S rRNA gene of Nnm. gari JCM14663 (AB545859), while the third one differs from the latter at two nucleotides. This indicates Natrinema sp. J7-2 is closely related to Nnm. gari. (TIF) [file pone.0041621.s002.tif]
