# Supplementary material for: The Complete Genome Sequence of Natrinema sp. J7-2, a Haloarchaeon Capable of Growth on Synthetic Media without Amino Acid Supplements
Source: PLoS One. 2012 Jul 23;7(7):e41621. doi: 10.1371/journal.pone.0041621 (PMC3402447; doi:10.1371/journal.pone.0041621)
Supplement: Table S1 — Predicted transporters in Natrinema sp. J7-2 genome. (DOC) [file pone.0041621.s003.doc]

**Table S1. Predicted transporters in *Natrinema* sp. J7-2 genome.**

| Function | Transporter | Component | | Gene |
| --- | --- | --- | --- | --- |
| Osmotic  regulation | Multicomponent K^+^/Na^+^:H^+^ antiporter | Pha/Mnh | A | 0878 |
|  |  |  | B | 1350, 1351 |
|  |  |  | C | 0879, 1349 |
|  |  |  | D | 0880, 1346, 1348 |
|  |  |  | E | 0881, 1354 |
|  |  |  | F | 0882, 1353 |
|  |  |  | G | 0883, 1352 |
|  | K^+^ uptake protein | Trk | A | 0077, 1107, 1110, 1022, 1328, 3766, 3901 |
|  |  |  | H | 0078, 1023, 1105, 1108 |
|  | Na^+^:H^+^ antiporter | TC.NhaC | | 1305, 1337, 4115, 1130, 1942 |
|  | Na^+^:solute symporter | TC.SSS | | 2484 |
|  | Mechanosensitive ion channel | MscS | | 3108 |
| Nutrient  uptake | Multiple sugar transport system | ABC. MS | A | 2959 |
|  |  |  | P | 2957, 2958 |
|  |  |  | S | 2956 |
|  | Simple sugar transport system | ABC. SS | A | 1483 |
|  |  |  | P | 1481, 1482 |
|  |  |  | S | 1484 |
|  | Multiple sugar transport system |  | | 2703, 2704, 2705, 2706, 2707 |
|  | Sugar transporter |  | | 0565, 0084 |
|  | Citrate transporter |  | | 0454 |
|  | Acetate transporter |  | | 3545 |
|  | Glycerol transporter |  | | 1712 |
|  | Peptide/nickel transporter | Opp | A | 0397, 1881, 1975, 2343, 3821 |
|  |  |  | B | 0398, 1882, 1976, 2341, 3820 |
|  |  |  | C | 0399, 0709, 1883, 1977, 2340, 3819 |
|  |  |  | D | 0400, 1885, 3818 |
|  |  |  | F | 0401, 0710, 0712, 1974, 2338, 3817 |
|  | Polar amino acid transporter | ABC. PA | A | 0506 |
|  |  |  | P | 0505, 0507 |
|  |  |  | S | 0503 |
|  | Basic amino acid/polyamine antiporter | TC.APA | | 1169 |
|  | Ammonium transporter | AmtB | | 0366 |
|  | Formate-nitrite transporter | FNT | | 3263 |
|  | Gluconate:H^+^ symporter | TC.GNTP | | 3288 |
|  | Sodium:proline symporter | putP | | 1874, 1953 |
|  | Glutamate/aspartate transport protein | GltP | | 0949 |
|  | Branched-chain amino acid transport system substrate-binding protein | livK | | 2769 |
|  | Cationic amino acid transporter |  | | 3467 |
|  | Amino acid ABC transporter |  | | 1242 |
|  | Amino acid transporter (UspA family) |  | | 2229, 2230 |
|  | oligopeptide transporter OPT family |  | | 3412 |
|  | Cyanate transport protein |  | | 2291 |
|  | Thiamine transporter | tbpA thiPQ | | 0450, 0451, 0452 |
|  | Sperimidine/putrescine transporter | potABC | A | 1054 |
|  |  |  | H | 1053 |
|  |  |  | C | 1052 |
|  |  |  | D | 1055 |
|  | Lipoprotein translocation system | Lol | C | 2112, 3863 |
|  |  |  | D | 2113, 3862 |
|  |  |  | E | 2111, 3864 |
|  | Lipid/cholesterol transport |  | | 0080 |
|  | ABC multidrug/lipid transport system |  | | 3922 |

**Table S1. Continued.**

| Function | Transporter | Component | | Gene |
| --- | --- | --- | --- | --- |
| Nutrient  uptake | Lipid/cholesterol transporter |  | | 1522 |
|  | Bile acid: Na^+^ symporter | TC.BASS | | 0615, 2744 |
|  | Sulfate transporter |  | | 2212, 2235 |
|  | Sulfonate/nitrate/taurine transporter | SsuABC | A | 1927 |
|  |  |  | C | 1928 |
|  |  |  | B | 1929 |
|  | Putative sulfate/molybdate transport |  | A | 2790 |
|  |  |  | B | 2791, 2792 |
|  |  |  | C | 2793 |
|  | Phosphate transporter | PstSCAB | A | 1585, 1592 |
|  |  |  | B | 1584, 1591 |
|  |  |  | C | 1586, 1593 |
|  |  |  | S | 1587, 1588, 1594 |
|  | Phosphate transport regulation protein | phoU | | 1582, 1596, 1590, 3622 |
|  | Phosphate transporter precursor | phoT | | 0420, 0502 |
|  | Inorganic phosphate | TC.PIT | | 0527, 0529 |
|  | Sodium-dependent phosphate transporter |  | | 3731 |
| Toxin  resistance | Antibiotic transporter | Yad | G | 0226, 0657, 2936 |
|  |  |  | H | 0227, 2937 |
|  | Multidrug resistance transport protein |  | | 0487 |
|  | Arsenite transporter | TC.ACR3 | | 0532 |
|  | Arsenical pump-driving ATPase | ArsA | | 0650, 4360, 1313 |
|  | Putative heavy metal transporter |  | | 0591, 0592, 0594, 0595 |
|  | Heavy metal transporting P-type ATPase |  | | 2579, 3091, 4100, 4232 |
| Cation  transport | Iron(III) transporter | AfuABC | A | 2192 |
|  |  |  | B | 2193 |
|  |  |  | C | 3128 |
|  | Iron complex transporter | Fhu | D | 2672, 2979 |
|  |  |  | B | 2157, 2978 |
|  |  |  | C | 2977 |
|  | Cobalt transporter | Cbi | Q | 1964 |
|  |  |  | O | 1965 |
|  | Zinc transporter | Znu | B | 1030, 3478 |
|  |  |  | C | 1031, 3479 |
|  |  |  | A | 1032, 3480 |
|  | Zinc transporter | TC.ZIP | | 2425 |
|  | Cation efflux system protein | TC.CDF | | 4135 |
|  | Ca^2+^:Cation antiporter | yrbG | | 3895, 4079 |
|  | Monovalent cation: H^+^ antiporter | TC.CPA1 | | 1830 |
|  | Manganese transport protein | mntH | | 0845, 2900 |
|  | Metal transporter | NRAMP | | 0835 |
|  | Cation diffusion facilitator |  | | 0272 |
|  | Na^+^/Ca^2+^ exchanging protein |  | | 2846 |
|  | Na^+^/Ca^2+^ antiporter | CaCA | | 4079 |
|  | Magnesium and cobalt efflux protein | CorC | | 0810 |
| Others | Neutrotransmitter | TC.NSS | | 0682, 3372 |
|  | Possible ABC transporter |  | | 3553, 3554, 3555 |
