# Supplementary material for: The Complete Genome Sequence of Natrinema sp. J7-2, a Haloarchaeon Capable of Growth on Synthetic Media without Amino Acid Supplements
Source: PLoS One. 2012 Jul 23;7(7):e41621. doi: 10.1371/journal.pone.0041621 (PMC3402447; doi:10.1371/journal.pone.0041621)
Supplement: Table S2 — Predicted enzymes involved in amino acid synthesis in Natrinema sp. J7-2. (DOC) [file pone.0041621.s004.doc]

**Table S2. Predicted enzymes involved in amino acid synthesis in *Natrinema*** sp. J7-2.

| EC | Abbr. | Enzyme | Genes ^a^ | AA ^b^ |
| --- | --- | --- | --- | --- |
| 1.4.1.3 | GDH | glutamate dehydrogenase (NAD(P)^+^) | 1599, 1939, 2174 | Glu |
| 1.4.1.4 | GDH | glutamate dehydrogenase (NADP^+^) | 3635 |
| 1.4.1.13/14 | GOGAT | glutamate synthase | 2607 |
| 6.3.1.2 | GS | glutamine synthetase | 1637, 2663 | Gln |
| 2.6.1.1 |  | aspartate transaminase | 1211, 3742 | Asp |
| 3.5.1.1 |  | L-asparaginase | 2851 |
| 6.3.5.4 |  | asparagine synthase | 3072 | Asn |
| 2.7.2.4 |  | aspartate kinase | 0474 | Lys  Thr  Met |
| 1.2.1.11 |  | aspartate-semialdehyde dehydrogenase | 0760 |
| 1.1.1.3 |  | homoserine dehydrogenase | 2409 |
| 2.7.1.39 | ThrB | homoserine kinase | 2473 | Thr |
| 4.2.3.1 | ThrC | threonine synthase | 2180 |
| 2.3.1.31 | MetX | homeserine O-acetyltransferase | 0561 | Met |
| 2.5.1.48 | MetB | cystathionine gamma-synthase | 1165 |
| 2.5.1.49 | MetY | O-acetylhomoserine (thilo)-lyase | 0562 |
| 2.1.1.14 | MetE | 5-methyltetrahydropteroyltriglutamate  -homocysteine methyltransferase | 1175,1176 |
| 4.2.1.52 | DapA | dihydrodipicolinate synthase | 4051 | Lys |
| 1.3.1.26 | DapB | dihydrodipicolinate reductase | 4050 |
| 2.6.1.17 | DapC | N-succinyldiaminopimelate aminotransferase | 0260 |
| 2.3.1.117 | DapD | 2,3,4,5-tetrahydropyridine-2-carboxylate N-succinyltransferase | 4049 |
| 3.5.1.18 | DapE | succinyl-diaminopimelate desuccinylase | 4045 |
| 5.1.1.7 | DapF | diaminopimelate epimerase | 4046 |
| 4.1.1.20 | LysA | diaminopimelate decarboxylase | 4047 |
|  | ArgX | putative glutamate--argW ligase | 0263 * | Arg |
|  | ArgW | putative biosynthetic carrier protein | 0264 * |
| 2.1.3.3 | ArgF/I | ornithine carbamoyltransferase | 0258 |
| 3.5.1.- | ArgE | acetyl-lysine deacetylase | 0259 |
| 2.6.1.11 | ArgD | acetylornithine | 0260 |
| 2.7.2.8 | ArgB | acetylglutamate kinase | 0261 |
| 1.2.1.38 | ArgC | N-acetyl-gamma-glutamyl-phosphate reductase | 0262 |
| 4.3.2.1 | ArgH | argininosuccinate lyase | 0265 |
| 6.3.4.5 | ArgG | argininosuccinate synthase | 0266 |
| 4.3.1.12 | Ocd | ornithine cyclodeaminase | 0781 | Pro |
| 1.2.1.41 | ProA | glutamate-5-semialdehyde dehydrogenase | 2084 |
| 2.7.2.11 | ProB | glutamate 5-kinase | 2085 |
| 1.5.1.2 | ProC | pyrroline-5-carboxylate reductase | 2086 |
| 1.5.99.8 | PutA | proline dehydrogenase | 1955 |
| 1.4.1.1 |  | alanine dehydrogenase | 0085 * | Ala |
| 2.4.2.17 | HisG | ATP phosphoribosyltransferase | 0587 | His |
| 3.6.1.31 | HisE | phosphoribosyl-ATP pyrophosphohydrolase | 2882 |
| 3.5.4.19 | HisI | phosphoribosyl-AMP cyclohydrolase | 0554 |
| 5.3.1.16 | HisA | phosphoribosylformimino-5-aminoimidazole carboxamide ribotide isomerase | 0524 |
| 4.1.3.- | HisF | cyclase HisF | 3024 |
| 2.4.2.- | HisH | glutamine amidotransferase | 1668 |
| 4.2.1.19 | HisB | imidazoleglycerol-phosphate dehydratase | 0521 |
| 1.1.1.23 | HisD | histidinol dehydrogenase | 2939 |
| 3.1.3.15 |  | histidinol-phosphatase | 3071 * |
| 2.6.1.9 | HisC | histidinol-phosphate aminotransferase | 0770, 1621 | His  Phe  Tyr |
| 3.1.3.3 | SerB | phosphoserine phosphatase | 4104, 0559 | Ser |
| 1.1.1.95 | SerA | D-3-phosphoglycerate dehydrogenase | 0762, 0555, 2895 |
|  |  | class V aspartate transaminases | 0571, 2948 |
| 1.1.1.81 |  | hydroxypyruvate reductase | 4038 |

**Table S2**. Continued.

| EC | Abbr. | Enzyme | Genes ^a^ | AA ^b^ |
| --- | --- | --- | --- | --- |
| 4.3.1.19 |  | serine/threonine dehydratase | 2201 | Ser  Ile |
| 2.1.2.1 | GlyA | glycine hydroxymethyltransferase | 3408 | Gly |
| 2.3.1.30 | CysE | serine O-acetyltransferase | 0429 | Cys |
| 2.5.1.47 | CysK | cysteine synthase A | 3435, 3600 |
| 2.8.1.1 |  | thiosulfate sulfurtransferase | 0294 * |
| 1.8.7.1 |  | sulfite reductase | 0304 * |
| 1.8.3.1 |  | sulfite oxidoreductase | 3031 * |
| 4.2.3.3 | MgsA | methylglyoxal synthase | 2096 | Trp  Phe  Tyr |
| 4.1.2.13 |  | fructose 1,6-bisphosphate aldolase | 3648 |
| 4.1.2.- |  | 2-amino-3,7-dideoxy-D-threo-hept-6-ulosonate synthase | 4134 * |
| 1.4.1.- |  | dehydroquinate synthase II | 4142 |
| 4.2.1.10 | AroD | 3-dehydroquinate dehydratase I | 4144 |
| 1.1.1.25 | AroE | shikimate 5-dehydrogenase | 4081 |
| 2.7.1.71 | AroB | shikimate kinase | 1533 |
| 2.5.1.19 | AroA | 3-phosphoshikimate 1-carboxyvinyltransferase | 3186 |
| 4.2.3.5 | AroC | chorismate synthase | 3604 |
| 4.2.1.20 | TrpA/B | tryptophan synthase | 4133/4132 | Trp |
| 4.1.1.48 | TrpC | indole-3-glycerol phosphate synthase | 4131 |
| 4.1.3.27 | TrpE | anthranilate synthase | 1565, 4083, 1566, 4084 |
| 2.4.2.18 | TrpD | anthranilate phosphoribosyltransferase | 0151, 1563 |
| 5.3.1.24 | TrpF | phosphoribosylanthranilate isomerase | 1564 |
| 5.4.99.5 | PheA1 | chorismate mutase | 1532 | Phe  Tyr |
| 2.6.1.1 | AspB | aspartate aminotransferase | 1211, 3742 |
| 4.2.1.51 | PheA2 | prephenate dehydratase | 0071 | Phe |
| 1.3.1.12 | TyrA | prephenate dehydrogenase | 3764 | Tyr |
| 2.6.1.42 | IlvE | branched-chain amino acid aminotransferase | 1686, 4092 | Leu  Val  Ile |
| 1.1.1.85 | LeuB | 3-isopropylmalate dehydrogenase | 1752 | Leu  Ile |
| 4.2.1.33 | LeuC/D | 3-isopropylmalate/(R)-2-methylmalate dehydratase | 1748/1749 |
| 2.2.1.6 |  | acetolactate synthase | 1744, 1949, 2312, 1745 | Ile  Val |
| 1.1.1.86 | IlvC | ketol-acid reductoisomerase | 1746 |
| 4.2.1.9 | IlvD | dihydroxy-acid dehydratase | 1237 |
| 2.3.3.13 | LeuA | 2-isopropylmalate synthase | 1743 | Leu |
| 2.3.1.182 | CimA | citramalate synthase | 1272 | Ile |

^a^ The putative homologous genes newly predicted in this study are indicated by stars.

^b^ The enzymes involved in the synthesis of the indicated amino acids.
