# Supplementary material for: The Complete Genome Sequence of Natrinema sp. J7-2, a Haloarchaeon Capable of Growth on Synthetic Media without Amino Acid Supplements
Source: PLoS One. 2012 Jul 23;7(7):e41621. doi: 10.1371/journal.pone.0041621 (PMC3402447; doi:10.1371/journal.pone.0041621)
Supplement: Table S3 — Alignments of some CRISPR spacers of Natrinema sp J7-2 with the most similar known sequences. (DOC) [file pone.0041621.s005.doc]

**Table S3. Alignments of some CRISPR spacers of *Natrinema* sp J7-2** with the most similar known sequences.

| Spacer | Source of known sequence | Alignment­­ ^a^ |
| --- | --- | --- |
| CRISPR1  Spacer 4 | *Sphingomonas wittichii* RW1  (Plasmid pSWIT02) | CAGGATGATGAGCAGGATGACGA  |||||||||||||||||||||||  CAGGATGATGAGCAGGATGACGA |
| CRISPR1  Spacer 32 | *Kitasatospora setae* KM-6054  (Chromosome) | CCGCTCGCGCTGGCGCTGGCTGTGGCT  |||||||||||||||||||||||||||  CCGCTCGCGCTGGCGCTGGCTGTGGCT |
| CRISPR1  Spacer 34 | *Vulcanisaeta moutnovskia*  (Chromosome) | GCTCAGGGTTGGTAGGGTCCTCGAGGT  |||| ||||||||||||||||||||||  GCTCCGGGTTGGTAGGGTCCTCGAGGT |
| CRISPR2  Spacer 1 | *Halorubrum lacusprofundi*  (Chromosome) | CGACGAGATCGAGGCGATCCTCGACGCCGGCG  ||||||| |||||||| || ||||||||||||  CGACGAGTTCGAGGCGTTCGTCGACGCCGGCG |
| CRISPR2  Spacer 2 | *Natrinema* sp. J7  (Plasmid pHH205) | TGAATATGGAGACTCGAGAGACTCGCCCTG  ||||||||| |||||||||||||| |||||  TGAATATGGGGACTCGAGAGACTCACCCTG |
| CRISPR3  Spacer 4 | *Frankia* sp. EuI1c  (Chromosome) | GTCGACGAGGTCCTCGAGATCG  ||||||||||||||||||||||  GTCGACGAGGTCCTCGAGATCG |

^a^ *Natrinema* sp J7-2 CRISPR spacer sequence (top line) and the best-match known sequence (bottom line).
